# Emerging variants, unique phenotypes, and transcriptomic signatures: an integrated study of *COASY* ‐associated diseases

**DOI:** 10.1002/acn3.52079

**Published:** 2024-05-15

**Authors:** Chiara Cavestro, Francesca Morra, Andrea Legati, Marco D'Amato, Alessia Nasca, Arcangela Iuso, Naomi Lubarr, Jennifer L. Morrison, Patricia G. Wheeler, Clara Serra‐Juhé, Benjamín Rodríguez‐Santiago, Eulalia Turón‐Viñas, Clement Prouteau, Magalie Barth, Susan J. Hayflick, Daniele Ghezzi, Valeria Tiranti, Ivano Di Meo

**Affiliations:** ^1^ Unit of Medical Genetics and Neurogenetics Fondazione IRCCS Istituto Neurologico Carlo Besta Milan Italy; ^2^ Institute of Human Genetics, School of Medicine Technical University of Munich Munich Germany; ^3^ Institute of Neurogenomics Helmholtz Zentrum München Neuherberg Germany; ^4^ Department of Neurology Icahn School of Medicine at Mount Sinai, Mount Sinai Beth Israel New York New York USA; ^5^ Division of Genetics, Arnold Palmer Hospital Orlando Florida USA; ^6^ Genetics Department Hospital de la Santa Creu i Sant Pau Barcelona Spain; ^7^ Centro de Investigación Biomédica en Red de Enfermedades Raras (CIBERER) Madrid Spain; ^8^ Genomic Instability Syndromes and DNA Repair Group and Join Research Unit on Genomic Medicine UAB‐Sant Pau Biomedical Research Institute Hospital de la Santa Creu i Sant Pau Barcelona Spain; ^9^ Child Neurology Unit, Pediatrics Service Hospital de la Santa Creu i Sant Pau Barcelona Spain; ^10^ Department of Genetics University Hospital of Angers Angers France; ^11^ Department of Molecular and Medical Genetics Oregon Health & Science University Portland Oregon USA; ^12^ Department of Pediatrics Oregon Health & Science University Portland Oregon USA; ^13^ Department of Neurology Oregon Health & Science University Portland Oregon USA; ^14^ Department of Pathophysiology and Transplantation University of Milan Milan Italy

## Abstract

**Objective:**

*COASY*, the gene encoding the bifunctional enzyme CoA synthase, which catalyzes the last two reactions of cellular *de novo* coenzyme A (CoA) biosynthesis, has been linked to two exceedingly rare autosomal recessive disorders, such as COASY protein‐associated neurodegeneration (CoPAN), a form of neurodegeneration with brain iron accumulation (NBIA), and pontocerebellar hypoplasia type 12 (PCH12). We aimed to expand the phenotypic spectrum and gain insights into the pathogenesis of *COASY*‐related disorders.

**Methods:**

Patients were identified through targeted or exome sequencing. To unravel the molecular mechanisms of disease, RNA sequencing, bioenergetic analysis, and quantification of critical proteins were performed on fibroblasts.

**Results:**

We identified five new individuals harboring novel *COASY* variants. While one case exhibited classical CoPAN features, the others displayed atypical symptoms such as deafness, language and autism spectrum disorders, brain atrophy, and microcephaly. All patients experienced epilepsy, highlighting its potential frequency in *COASY*‐related disorders. Fibroblast transcriptomic profiling unveiled dysregulated expression in genes associated with mitochondrial respiration, responses to oxidative stress, transmembrane transport, various cellular signaling pathways, and protein translation, modification, and trafficking. Bioenergetic analysis revealed impaired mitochondrial oxygen consumption in *COASY* fibroblasts. Despite comparable total CoA levels to control cells, the amounts of mitochondrial 4′‐phosphopantetheinylated proteins were significantly reduced in *COASY* patients.

**Interpretation:**

These results not only extend the clinical phenotype associated with *COASY* variants but also suggest a continuum between CoPAN and PCH12. The intricate interplay of altered cellular processes and signaling pathways provides valuable insights for further research into the pathogenesis of *COASY*‐associated diseases.

## Introduction

Coenzyme A (CoA) is an indispensable and ubiquitous cofactor required for a myriad of enzymatic activities and cellular processes, which is synthesized from pantothenic acid (vitamin B5), cysteine, and ATP via a highly conserved five‐step pathway.^1^ *COASY*, located on chromosome 17q21.2, encodes the 564 amino acids bifunctional enzyme 4′‐phosphopantetheine adenylyltransferase (PPAT)/dephospho‐CoA kinase (DPCK), also known as CoA synthase, which catalyzes the last two steps of the CoA biosynthetic pathway.^2^ COASY is mainly localized in mitochondria, although cytoplasmic and nuclear localization has been reported.^3^, ^4^, ^5^, ^6^, ^7^

Recessive COASY pathogenic variants cause COASY protein‐associated neurodegeneration (CoPAN), a rare disorder in the neurodegenerations with brain iron accumulation (NBIA) group.^6^ It typically manifests with oro‐mandibular and limb spasticity, dystonia, spastic‐dystonic tetraparesis, and parkinsonisms. Psychiatric disorders like obsessive‐compulsive and depression may also occur. Patients with this condition have brain MRI findings,^6^, ^8^, ^9^ indicating iron deposition, similar to those seen in pantothenate kinase‐associated neurodegeneration (PKAN), which consists of bilateral hyperintensity with surrounding hypointensity in the *globus pallidus* (GP) in T2‐weighted images (“eye of the tiger sign”). PKAN is the most common NBIA subtype, caused by variants in the mitochondrial pantothenate kinase 2 gene (*PANK2*), which catalyzes the first step in the *de novo* CoA biosynthetic pathway.^1^

Notably, complete loss‐of‐function *COASY* variants have been identified in a total of 13 fetuses/newborns with pontocerebellar hypoplasia type 12 (PCH12), which is characterized by a fatal perinatal outcome defined by pons and cerebellar atrophy, microcephaly, and arthrogryposis, but without evidence of brain iron accumulation.^10^, ^11^ However, homozygous nonsense variant has recently been reported in a 21‐year‐old individual with spastic paraparesis, dysarthria, dysphagia, parkinsonism, and mild cerebellar atrophy and bilateral mineralization in GP.^12^

Recently, cases reported intermediate clinical phenotypes with new features. Two patients had hypotonia and respiratory failure, linked to brainstem and basal ganglia atrophy without iron overload.^13^ A mild form of CoPAN was reported in an adult patient with speech delay, gait disturbance, mild hypokinesia, and dystonic posture.^14^ Finally, a 6‐year‐old patient was reported with developmental delay and status epilepticus, associated with microcephaly, facial dysmorphism, basal ganglia, and thalamus T2 hyperintensities, thinning of the corpus callosum, and *globus pallidus* calcification.^15^ Remarkably, biallelic *COASY* variants have recently been found in 16 Chinese individuals with riboflavin‐responsive lipid storage myopathy (RR‐LSM), a subtype of lipid metabolism disorder characterized by muscle weakness or exercise intolerance, accumulation of lipid droplets in myofibers, and responsiveness to riboflavin.^16^ The extreme variability of the clinical features therefore raises the possibility that there are unrecognized symptoms associated with genetic variations in this gene.

Here, we report a detailed clinical and molecular description of five new cases from four unrelated families carrying four novel *COASY* variants and displaying classic or previously unreported clinical features. In addition, RNA‐seq analysis in the patients' skin fibroblasts identifies specific transcriptional changes impacting cellular processes, which may provide novel insights into the pathogenesis of *COASY*‐associated diseases.

## Materials and Methods

### Genetic analysis

Exome sequencing (ES) or custom clinical ES (CES) were performed in different centers using genomic DNA and published procedures.^17^, ^18^ Variants were confirmed by Sanger sequencing.

American College of Medical Genetics and Genomics (ACMG) classification^19^ of new variants was carried out starting from those present in Varsome (https://varsome.com) and Franklin (https://franklin.genoox.com) and adjusted according to the findings of this study. Variants pathogenicity was predicted using the public tools SIFT,^20^ Polyphen‐2,^21^ MutationTaster,^22^ and CADD.^23^ Protein normal mode analysis was carried out with DynaMut,^24^ using Alphafold predicted COASY structure (Q13057).

### Cell culture

Primary fibroblasts were cultured in high glucose Dulbecco's Modified Eagle Medium (Euroclone) supplemented with 10% fetal bovine serum (Euroclone), 1% penicillin/streptomycin (Euroclone), and 4 mM glutamine (Euroclone), at 37 °C in a 5% CO_2_ humidified atmosphere. Control fibroblasts were derived from normal individuals, and skin biopsies were taken at 25–30 years of age.

### Real‐time quantitative PCR

Total RNA was isolated from fibroblasts with the RNeasy Mini Kit (QIAGEN). Using the GoScript Reverse Transcriptase kit (Promega), RNA was reverse transcribed into cDNA, and qPCR was performed in duplicate using the iTaq Universal SYBR Green Supermix (Bio‐Rad). The amounts of target cDNA were compared using the ∆∆Cq method, relative to the beta‐actin (*ACTB*) gene.

### Immunoblotting

Sample preparation and SDS‐PAGE were performed as described,^25^ using the following primary antibodies: anti‐COASY (Thermofisher, PA528696), anti‐GAPDH (Millipore, MAB374), anti‐NDUFAB1 (Origene, TA308227), and anti‐ALDH1L2 (Proteintech 21391‐1‐AP).

### RNA sequencing and analysis

Total RNA was processed following the TruSeq Stranded mRNA protocol (Illumina). Libraries were then sequenced on the NextSeq550 platform (Illumina), and generated FASTQ files were used as input for Salmon to calculate raw counts per gene. Quality control, principal component analysis (PCA), and differential gene expression were performed with iDEP 1.1.^26^ Read count data were normalized by counts per million function in edgeR, transformed using the logarithm function, and used for gene expression analysis based on DESeq2 package, applying a cutoff of a false discovery rate (FDR) ≤0.01 and a log_2_ fold change ≥1. Gene ontology (GO) biological process and KEGG pathway enrichment analysis were performed using ShinyGO.^27^ Canonical pathway enrichment was carried out with Ingenuity Pathway Analysis (IPA) software (QIAGEN). Protein–protein interaction (PPI) network and Markov Cluster (MCL)‐based analysis were performed using String (https://string‐db.org) and Cytoscape.

### Determination of respiratory activity

The oxygen consumption rate (OCR) was measured on fibroblasts with an XF96 Extracellular Flux Analyzer (Agilent) as described.^28^

### Measurement of cellular CoA

Total cellular CoA was measured using a fluorimetric‐based kit (Abcam), according to manufacturer instructions as described.^29^

### Statistical analysis

Data were analyzed with GraphPad Prism 9, using two‐tailed unpaired Student's *t*‐test or one‐way ANOVA followed by Dunnett's post hoc test. The data are reported as the mean ± SD. The *p*‐value <0.05 was considered statistically significant.

## Results

### Clinical presentations

**Figure 1:**
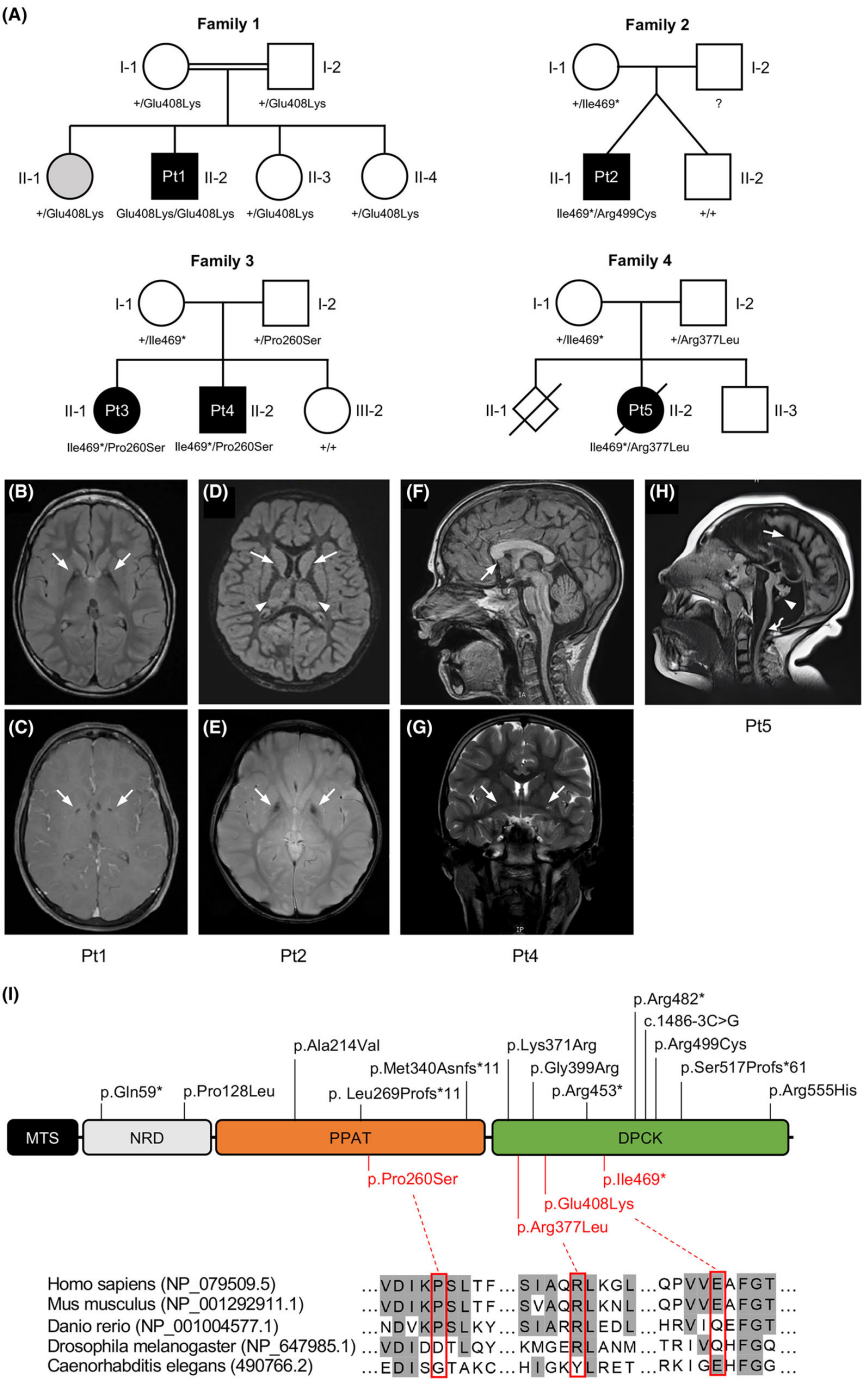
Genetics and MRI of subjects affected by variants in *COASY*. (A) Pedigree of the four families showing the segregation of alleles. The variants status of affected and unaffected subjects is indicated by black and white symbols, respectively. Gray color in subject II‐1 from family 1 indicates an individual affected by Friedreich's Ataxia, but heterozygous for *COASY* variant. Genotype of subject I‐2 from family 2 is not available. (B–H) Brain MRI of affected subjects. (B) Bilateral T2 hyperintensity within the anteromedial region (arrows), and (C) small round area of T1 hypointensity in the center of the globus pallidus (arrows) in Pt1 at the age of 12 years. (D) T2‐weighted FLAIR hyperintensities within the bilateral basal ganglia and thalamic nuclei (arrows), and (E) T1 hypointensity in the center of the globus pallidus (arrows) in Pt2 at the age of 3 years. (F) MRI showing rostrum of the corpus callosum agenesis (arrow) and (G) prominent anterior commissure (arrows) in sagittal T1‐weighted and coronal T2‐weighted MRI, respectively, from Pt4 at 10 years old. (H) Sagittal T2‐weighted FLAIR from Pt5 at 5 years old showing a massive cerebral atrophy (arrow), marked cerebellum and pons atrophy (arrowhead), and significant trunk atrophy (curved arrow). (I) Schematic representation of COASY protein with functional regions, novel variants identified in this study (red), and already reported variants (black). DPCK, dephospho‐CoA kinase; MTS, mitochondrial targeting sequence; NRD, N‐terminus regulatory domain; PPAT, 4′‐phosphopantetheine adenylyltransferase. Amino acid sequence alignment showing conservation degree of novel missense variants across species.

The main clinical findings of the patients are summarized in Table 1. Family pedigrees are shown in Figure 1A.

**Table 1:** Genetic, clinical, and neuroradiological features.

	Family 1	Family 2	Family 3		Family 4
Member	II‐2	II‐1	II‐1	II‐2	II‐2
Individual	Pt1	Pt2	Pt3	Pt4	Pt5
*COASY* variants (NM_025233.7)	c.1222G>A p.(Glu408Lys)	c.1403_1404dup p.(Ile469Ter); c.1495C>T p.(Arg499Cys)	c.1403_1404dup p.(Ile469Ter); c.778C>T p.(Pro260Ser)	c.1403_1404dup p.(Ile469Ter); c.778C>T p.(Pro260Ser)	c.1403_1404dup p.(Ile469Ter); c.1130G>T p.Arg377Leu
Zigosity	Homozygous	Compound heterozygous	Compound heterozygous	Compound heterozygous	Compound heterozygous
Consanguinity	+	−	−	−	−
Sex	M	M	F	M	F
Age at onset	16 months	14 months	2 years	Neonatal	Prenatal
Current age/death	22 years	4 years	13 years	12 years	Death at 7 years
Epilepsy	+	+	+	+	+
Developmental delay	−	+	−	+	−
Weakness	+	−	−	−	+
Hypotonia	+	−	−	−	−
Dystonia	+	−	−	−	−
Cognitive decline	+	−	−	−	−
Hearing loss	−	+	+	+	+
Speech delay	−	+	+	+	−
Strabismus	−	−	+	+	−
Pigmentary retinopathy	−	−	−	−	+
Autism	−	−	−	+	−
Hepatic abnormalities	−	+	−	−	−
Renal lithiasis	−	−	−	−	+
MRI	Increased T2 signal in globus pallidus (eye of the tiger sign)	Increased T2 signal in caudate nuclei, putamen, thalami; decreased cortical gray, and white matter volume	Normal until 3 years (cochlear implant placement)	Dysgenesis of the corpus callosum, prominence of the anterior commissure	Pontocerebellar hypoplasia, cerebral and cerebellum trunk atrophy, microcephaly
Brain iron accumulation	+	+	−	−	−

Pt1 (II‐2, family 1) is a 22‐year‐old male born without complications to healthy, consanguineous American parents of Italian descent. One sister has Friedreich's ataxia. Starting at 16 months, he showed an awkward gait and falls, slowed head circumference growth, hypotonia, and motor/speech delays. Neurological symptoms, including weakness, dystonia, and cognitive decline, appeared around the age of 5, and by age 13, he was wheelchair dependent. He had a single seizure at the age of 7. No further crises until the age of 19, when three seizure episodes manifested over 3 months, and a treatment with levetiracetam started with good seizure control. After 1 year, new seizures occurred, requiring an increase in the levetiracetam dose. An MRI at 12 years revealed pallidal iron deposition with the eye of the tiger sign (Fig. 1B,C).

Pt2 (II‐1, family 2) is a 4‐year‐old boy from the US, born at 35 weeks of gestation from a spontaneous twin pregnancy. His male twin had normal development. He developed seizures at 14 months. Subsequently, absence seizures and a possible focal seizure occurred. EEG was unremarkable. Brain MRI showed restricted diffusion involving bilateral caudate nuclei, putamen, and thalami with mild swelling and T2, and flair hyperintense signal changes without contrast enhancement (Fig. 1D,E). A reduced volume of cortical gray and white matter was suspected. Abdominal ultrasound showed a liver above normal size for age, with mild diffuse increased echotexture. Early development was delayed; walking began at 16 months. Speech is limited to two words and a few signs. The patient receives speech, occupational, and physiotherapy. Ear tubes were inserted due to failed hearing tests.

Patient 3 (Pt3; II‐1, family 3) is a 13‐year‐old Spanish girl who was diagnosed with bilateral profound hearing loss due to speech delay at the age of 2. She was fitted with a left cochlear implant at the age of 4. The patient also suffered from strabismus. Brain MRI and neurological evaluation before implantation were normal. At the age of 6, she was diagnosed with a language development disorder. At 10 years old, she presented with seizures. An EEG was performed, showing multifocal spikes and spike‐and‐wave complexes. She started treatment with oxcarbazepine and levetiracetam, with good seizure control.

Her 12‐year‐old brother (Pt4; II‐2, family 3) showed psychomotor retardation and started walking at 27 months. He also had strabismus and speech delay, but unlike his sister, he also showed social and communication difficulties. At the age of 2, already with suspected autism, he was diagnosed by tone audiometry with severe sensorineural hearing loss in the right ear and moderate hearing loss in the left ear. An MRI was performed, which showed dysgenesis of the corpus callosum (Fig. 1F,G). Despite two hearing aids, he was unable to develop his speech properly and was eventually diagnosed with an autism spectrum disorder. At 8 years old, he developed focal seizures, with the same semiology as his sister. He was initially treated with oxcarbazepine and levetiracetam, but due to his severe irritability, levetiracetam was replaced with perampanel, which led to good seizure control.

Pt5 (II‐2, family 4) was a young French girl born following a second pregnancy after the first was medically terminated due to cerebellar hypoplasia. Her prenatal period exhibited PCH, massive cerebral and cerebellar atrophy, and microcephaly. A slight decrease in motor and sensory nerve conduction velocities, myotonic bursts, fibrillations, and slow potentials were recorded. She developed severe epileptic encephalopathy and occasional choreoathetoid movements. At the age of 5, an MRI confirmed persistent prenatal alterations (Fig. 1H). Gastrostomy was necessary for feeding, and a constant state of pulmonary congestion required O_2_ supplementation. Renal lithiasis and scoliosis characterized the clinical picture on a kidney and orthopedic level. The electroretinogram revealed impaired vision with pigmentary retinopathy, and auditory evoked potentials indicated a distinct V wave decrease linked to cerebellum trunk pathology. Mitochondrial respiratory complex activities in skeletal muscle were reduced (complex I: 20%, complex III: 70%, complex IV: 50%), with severe muscle histology showing immature differentiation and a prevalence of type 2 fibers. Metabolic investigations showed hyperlactacidemia, increased lactate and pyruvate levels, global hypoaminoacidemia in plasmatic acid chromatography, and hyperaminoaciduria with elevated glycine levels in urinary acid chromatography, likely associated with sodium valproate treatment. Death occurred at the age of 7.

### Genetic studies

Details of the identified variants in *COASY* are provided in Table 1. All variants are numbered according to the NM_025233.7 reference sequence.

In Pt1, due to the presence of a typical MRI, a targeted genetic test for *PANK2* was performed, without finding any variant. Then, *COASY* sequencing revealed a novel missense variant, c.1222G>A (p.Glu408Lys) in a homozygous state, segregating within family members (Fig. 1A). The variant, located in the DPCK domain (Fig. 1I), is absent in gnomAD, and the PhyloP conservation score at the variant site is 1.265. According to CADD (Phred score of 20.4), this variant is predicted to possess a highly deleterious potential. However, SIFT, Polyphen‐2, and MutationTaster predicted it as benign. According to the ACMG guidelines, the variant can be classified as VUS (BP4, PM2_supporting).

In Pt2, ES found two heterozygous variants in the DPCK domain of *COASY*. The c.1403_1404dup (p.Ile469Ter) nonsense variant was found to be maternally inherited, while the c.1495C>T (p.Arg499Cys) missense variant is of unknown inheritance since the father was not available for testing (Fig. 1A). While the p.Arg499Cys is the most frequent pathogenic variant (PM3, PP3, PM2_supporting, PS3) associated with CoPAN,^6^, ^8^, ^9^, ^14^ the novel p.Ile469Ter truncating variant is not reported in population variant databases and was classified as pathogenic (PVS1, PM2_supporting, PM3) according to the ACMG criteria.

In Pt3, CES identified two heterozygous variants in the *COASY* gene: the nonsense c.1403_1404dup (p.Ile469Ter), the same found in Pt2, and a novel missense c.778C>T (p.Pro260Ser). Sanger sequencing in Pt4 revealed the presence of the variants, both segregating within the family (Fig. 1A). The p.Pro260Ser is located in the PPAT domain (Fig. 1I), and nonsynonymous variants are rare in this region; furthermore, it is very rare in the gnomAD database (frequency = 0.0000263). PhyloP conservation score at the site variant is 7.54, and the CADD Phred score of 25.2 suggested its deleteriousness. SIFT, Polyphen‐2, and MutationTaster predicted it as benign, possibly damaging, and uncertain, respectively, while its germline ACMG classification was likely pathogenic (PM1, PM3, PM2_supporting, PP3).

In Pt5, due to multiple mitochondrial respiratory complexes defects and the ophthalmic phenotype, the complete mtDNA sequencing and a hereditary optic neuropathy NGS panel were carried out, with no evidence of pathogenic variants. Then, ES identified the already mentioned *COASY* variant c.1403_1404dup (p.Ile469Ter) in trans with a novel missense c.1130G>T (p.Arg377Leu), both segregating within the family. The p.Arg377Leu is located in the DPCK domain (Fig. 1I), is not reported in any population variant database, and was classified as VUS (BP4, PM2_supporting, PM3). PhyloP conservation score at the site variant is 1.444. SIFT, Polyphen‐2, and MutationTaster predicted it as benign, possibly damaging, and benign, respectively, while CADD suggested its deleteriousness (Phred score of 21.2).

### Variants characterization

**Figure 2:**
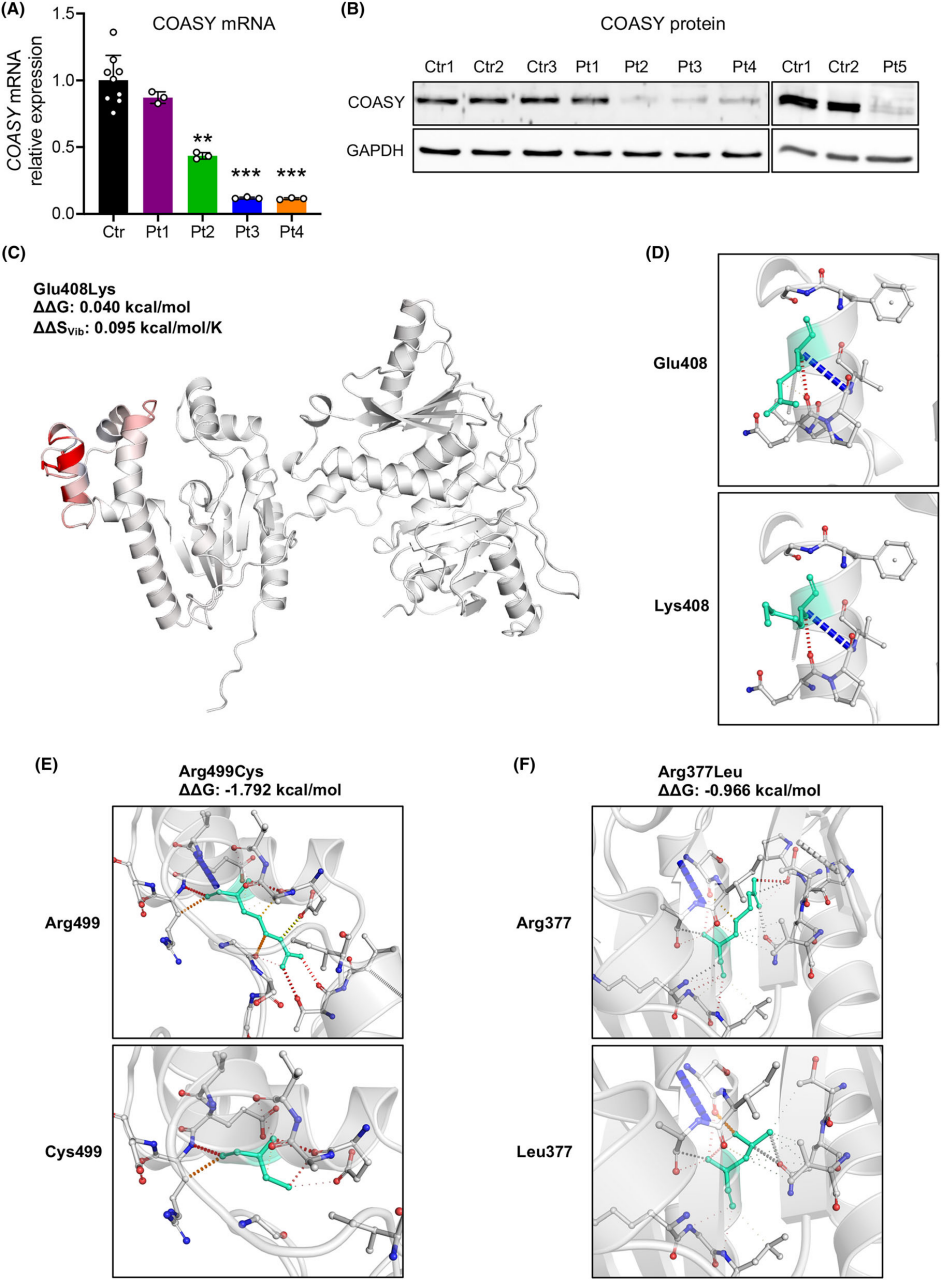
Effects of identified variants. (A) qPCR analysis showing relative *COASY* mRNA expression in control (Ctr, *n* = 3) and patients (Pt) fibroblasts. Mean of three independent experiments ± SD is shown. **p < 0.01; ***p < 0.001 (one‐way ANOVA). (B) Immunoblot analysis of COASY protein in fibroblast from controls (Ctr) and patients (Pt). An antibody against GAPDH was used as control. (C) Changes in vibrational entropy energy caused by Glu408Lys variant. Red portion represents a gain in flexibility upon mutation. The predicted change in free energy (ΔΔG) and vibrational entropy (ΔΔS_Vib_) is shown. (D–F) Analysis of contact mediated by residues around mutation sites for (D) Glu408Lys, (E) Arg499Cys, and (F) Arg377Leu. Wild‐type (up) and variant (down) residues are colored in turquoise. H‐bonds are shown in red, Van der Waals (VdW) contacts in gray, hydrophobic‐VdW clashes in green, and polar‐VdW clashes in orange.

qPCR showed that *COASY* transcript was reduced in skin fibroblasts to about 50% in Pt2 and to 10% in Pt3 and Pt4, while in Pt1 it was comparable to that of control cells (Fig. 2A). RNA from Pt5 was not available for testing. Similarly, immunoblot analysis revealed a severe reduction of the protein amount in fibroblasts from Pt2, Pt3, Pt4, and Pt5 and the presence of nearly normal protein content in Pt1 (Fig. 2B).

These data indicate that in Pt1, the p.Glu408Lys change does not affect the amount of either the transcript or protein. Normal mode‐based analysis of protein 3D structure showed that the p.Glu408Lys does not alter protein stability (ΔΔG: 0.040 kcal/mol), but can induce an increase in vibrational entropy (ΔΔS_Vib_: 0.095 kcal/mol/K), which is linked to a gain of molecular flexibility (Fig 2C), while no drastic changes are observed in interatomic interactions (Fig. 2D).

In Pt2, the reduction at about half of *COASY* transcript, as well as the minimal amount of the protein, suggest a mechanism of nonsense‐mediated mRNA decay for the p.Ile469Ter variant, while the p.Arg499Cys was confirmed to be associated with protein instability.^6^ In fact, the p.Arg499Cys variant is predicted to induce a significant protein destabilization (ΔΔG: −1.792 kcal/mol), and a drastic change of the structural conformation, due to a loss of several atomic interactions between the residue in position 499 and the adjacent side chain (Fig. 2E).

Conversely, the missense p.Pro260Ser variant found in trans with the nonsense p.Ile469Ter in Pt3 and Pt4 is associated with the severe decrease of *COASY* transcript, suggesting a mechanism of mRNA decay, reclassifying the variant as pathogenic (PS3 in addition to PM1, PM2_supporting, PM3 and PP3). Finally, the p.Arg377Leu variant, again found in trans with the p.Ile469Ter in Pt5 and associated with severe reduction of the protein in fibroblasts, could causes either mRNA decay or protein instability (Fig. 2F), changing its classification to likely pathogenic (PS3 in addition to BP4, PM2_supporting, PM3).

### Transcriptomic analysis

**Figure 3:**
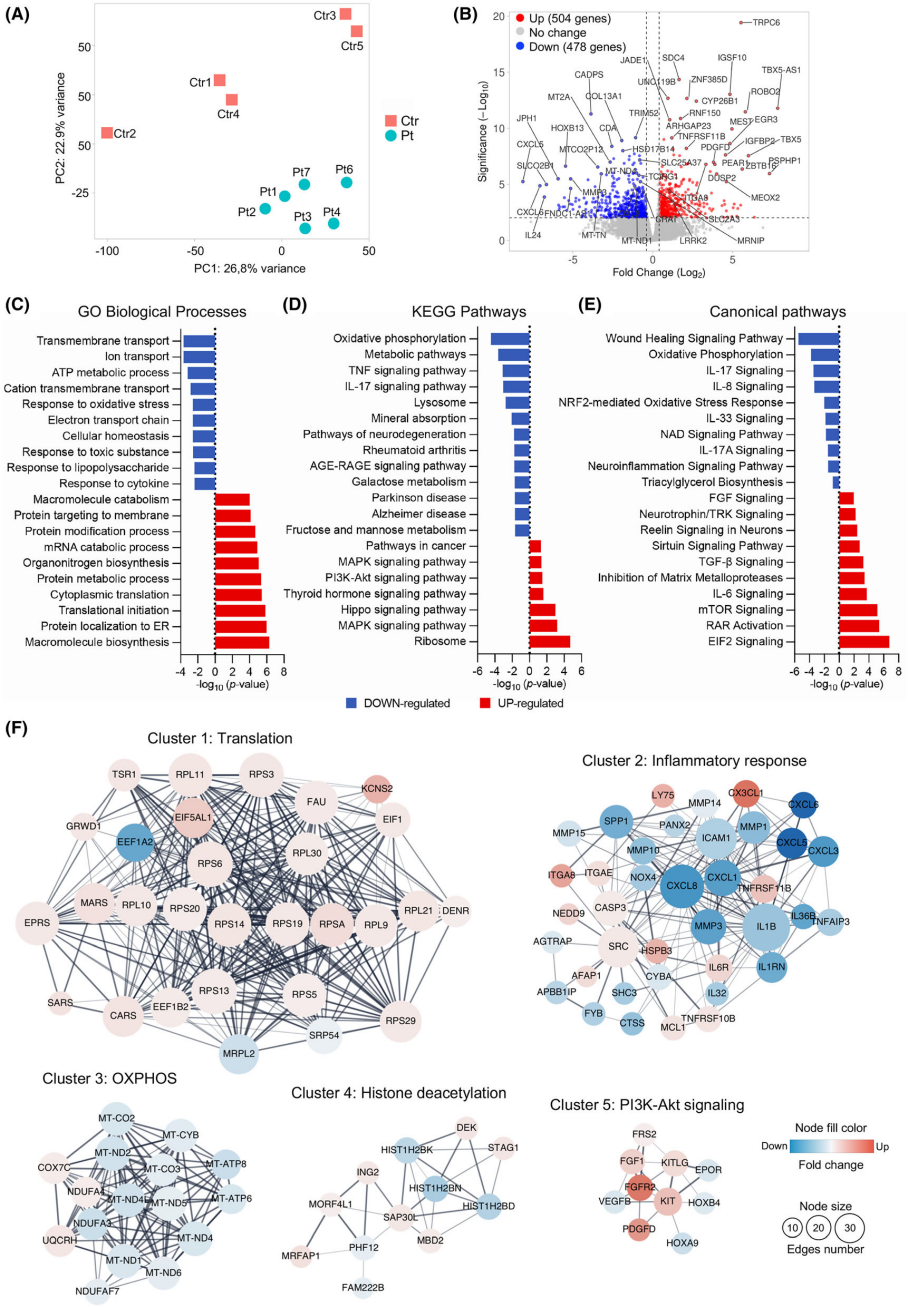
Transcriptomic analysis in fibroblasts. (A) Principal component analysis (PCA) plot of RNA‐seq data, showing clear separation of patient‐derived fibroblast (turquoise) from controls (red). The two axes PC1 and PC2 represent the first two principal components identified by the analysis. (B) Volcano plots of the calculated DEGs. The number of the up‐regulated (red) and down‐regulated (blue) is indicated for log_2_FC >1 and *p*‐value <0.001. Top 50 DEGs are highlighted. (C–E) Selection of the most significantly enriched (C) GO biological process, (D) KEGG pathways, and (E) canonical pathways of DEGs, expressed as −log_10_ (*p*‐value). Down‐ and up‐regulated processes and pathways are colored in blue and red, respectively. (F) Results of protein–protein interaction (PPI) network analysis of common DEGs. The five top‐scored clusters. Red circles represent up‐regulated genes, and blue circles represent down‐regulated genes. The color depth represents the fold change of hub genes. The size of the nodes indicates the connections number of each gene.

To investigate the potential impact of *COASY* variants on global gene expression, we performed an unbiased bulk transcriptomic study in fibroblasts from five healthy control individuals (Ctr1‐5), four of the individuals described above (Pt1‐4), and the two originally described patients with *COASY* variants (Pt6 and Pt7).^6^ We found detectable gene expression of 17,155 annotated transcripts. PCA showed that the control and patient groups were well separated based on the expression of the significantly altered genes (Fig. 3A). Gene expression analysis identified 982 differentially expressed genes (DEGs), of which 504 were up‐regulated and 478 were down‐regulated in *COASY* patients compared to control cells (Fig. 3B).

To gain insight into the molecular mechanisms involved in *COASY*‐associated disorders, we used the separate lists of up‐ or down‐regulated DEGs as input to perform statistical over‐representation tests. Gene Ontology (GO) enrichment analysis of biological processes revealed that the down‐regulated transcripts are mainly associated with ions and solutes transmembrane transport, cellular homeostasis, ATP‐dependent metabolic processes, mitochondrial electron transport chain, response to oxidative and toxic stress, and response to inflammatory cytokines (Fig. 3C). In addition, KEGG pathway enrichment analysis showed that the down‐regulated genes are associated with oxidative phosphorylation (OXPHOS) and metabolic pathways, inflammatory response, lysosomes, mineral absorption, and neurodegeneration (Fig. 3D). On the other hand, up‐regulated DEGs are enriched in biological processes mainly associated with protein biosynthesis, modification, and trafficking, (Fig. 3C), as well as in pathways involved in ribosomal activity, and MAPK, Hippo, thyroid hormone, and PI3K‐Akt signaling pathways (Fig. 3D).

Canonical pathway enrichment showed that up‐regulated pathways are linked to EIF2, retinoic acid receptor (RAR), mTOR, IL‐6, and matrix metalloproteases, while pathways involved in wound healing signaling, OXPHOS, production of different interleukins, NRF2‐mediated oxidative stress response, NAD signaling, and triacylglycerol biosynthesis were down‐regulated (Fig. 3E).

The full list of DEGs was used to construct a PPI network and perform an MCL‐based analysis. The top five identified cluster subnetworks are shown in Figure 3F. GO enrichment analysis of protein composing each subnetwork revealed that cluster 1 was associated with protein translation, cluster 2 was functionally related to the response to inflammation, cluster 3 was involved in mitochondrial respiration, cluster 4 was related to histone deacetylation, and cluster 5 was associated with PI3K‐Akt signaling.

### Functional investigations

**Figure 4:**
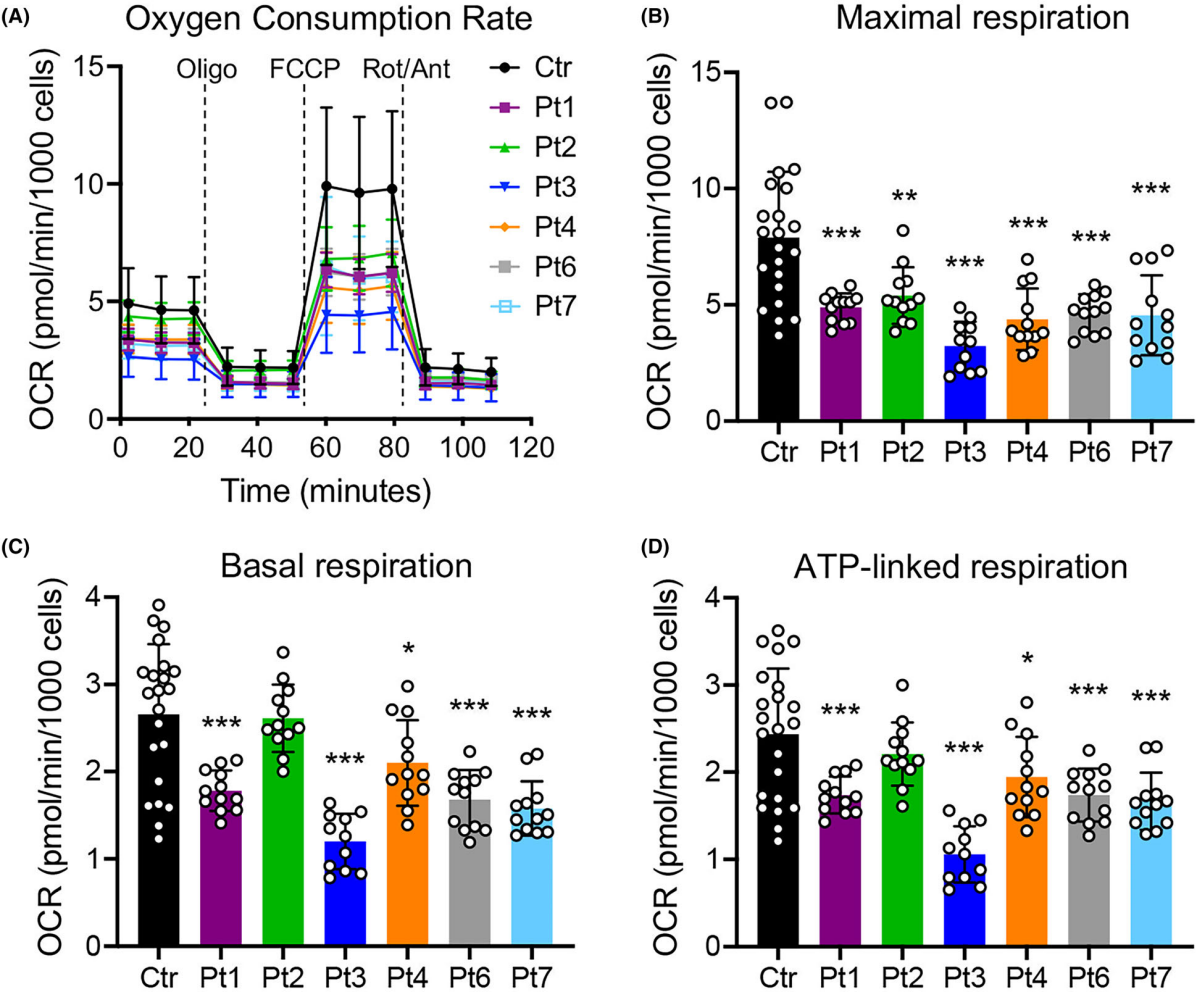
Respiration profile of COASY mutated fibroblasts. (A) OCR of fibroblasts, expressed as pmoles O_2_/min/1000 cells, under basal conditions and after injection of oligomycin (Oligo), carbonyl cyanide 4‐(trifluoromethoxy) phenylhydrazone (FCCP), rotenone (Rot) and antimycin A (Ant). Data are shown as mean ± SD of three control lines (Ctr, *n* = 22) and patients' cells (Pt, *n* = 12). (B–D) Maximal (B), basal (C), and ATP‐linked (D) respiration were calculated from OCR traces and are reported in the graphs as mean ± SD. *p < 0.05; **p < 0.01; ***p < 0.001 (one‐way ANOVA).

**Figure 5:**
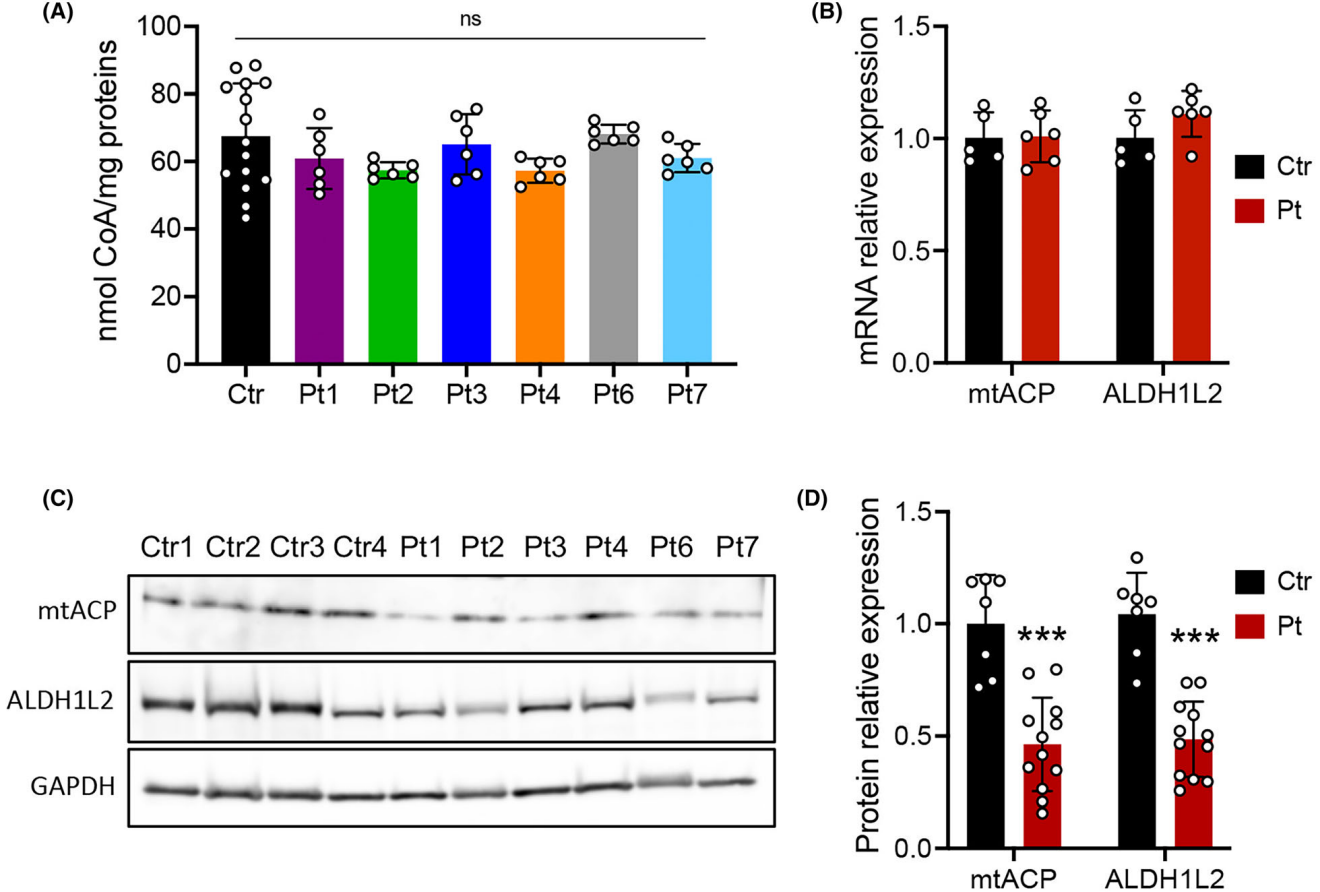
Analysis of CoA and mitochondrial 4′‐phosphopantetheinyl proteins in COASY mutated fibroblasts. (A) Total cellular CoA measured in three control lines (Ctr, *n* = 15) and patients (Pt, *n* = 6) fibroblasts expressed as nmol/mg of proteins. Data are shown as mean ± SD. *p < 0.05; **p < 0.01; ***p < 0.001 (one‐way ANOVA). (B) Relative amount of mtACP and ALDH1L2 transcripts assessed by qPCR. (C) Representative immunoblot and (D) densitometric quantification of mtACP and ALDH1L2 proteins in fibroblast from controls (Ctr) and patients (Pt) derived from two independent experiments. An antibody against GAPDH was used as control. Mean ± SD is shown. ***p < 0.001 (unpaired Student's *t*‐test).

OXPHOS genes were consistently identified as down‐regulated in the RNAseq data analysis. We evaluated OCR in our cohort of patients' fibroblasts and observed an overall impaired mitochondrial respiration (Fig. 4A). All patients showed a significant decrease in maximal respiration (Fig. 4B), while all except Pt2 displayed a defect in both basal (Fig. 4C) and ATP‐linked (Fig. 4D) respiration. Although *COASY* is the only known gene encoding for a CoA synthase enzyme, we surprisingly found that RNAseq analysis did not detect differentially expressed genes in pathways related to CoA metabolism. We previously showed that CoA levels were normal in fibroblasts derived from the two individuals originally described with *COASY* variants.^6^ Here, we again measured total cellular CoA levels in *COASY* fibroblasts, revealing normal CoA levels in all affected individuals (Fig. 5A), thus confirming previous data.

In most CoA‐dependent reactions, CoA serves as acyl carrier, transferring the acyl moiety between molecules without consuming CoA. 4'‐Phosphopantetheinylation (4PPTylation) of proteins is a post‐translational modification representing the only CoA‐consuming reaction.^30^ Two mitochondrial proteins undergo 4PPTylation: the mitochondrial acyl carrier protein (NDUFAB1, also known as mtACP), which is also an accessory subunit of the respiratory chain complex I,^31^ and the 10‐formyltetrahydrofolate dehydrogenase (ALDH1L2), involved in the maintenance of mitochondrial integrity and energy balance of the cell.^32^ Although no changes in the transcript levels were observed in the transcriptomic datasets confirmed by qPCR (Fig. 5B), we found that both mtACP and ALDH1L2 protein levels were significantly reduced in *COASY* fibroblasts (Fig. 5C,D).

## Discussion

Depending on the effect on the stability of the protein, pathogenic variants in *COASY* have been associated with two distinct clinical presentations, namely CoPAN and PCH12. However, the full phenotypic spectrum of *COASY*‐associated diseases is probably still unknown because so few cases have been identified. Nevertheless, a recent lifetime risk gnomAD analysis ranks CoPAN as the third most common NBIA disorder, presenting a global allele frequency of COASY pathogenic variants at 0.0012 and a lifetime risk of 0.15 per 100,000.^33^

We described here five individuals from four unrelated families who carry four novel variants in the *COASY* gene and show a broad spectrum of clinical features. While Pt1 had most of the typical CoPAN features, the remaining subjects had a combination of features and symptoms that are not yet described for *COASY*‐associated diseases. Seizures were reported for all the described patients. To date, only one report has described a status epilepticus associated with *COASY* variants.^15^ Our data suggest that epilepsy may be a common clinical feature of *COASY*‐associated diseases. Pt3 and Pt4, siblings from family 3, and Pt5 were characterized by hearing loss. Both Pt3 and Pt4 also had strabismus, while Pt4 was diagnosed with an autism spectrum disorder. Pt5, who had the most severe phenotype, was also affected by pigmentary retinopathy, neuromuscular involvement, multiple mitochondrial complex defects in muscle, and hyperlactacidemia.

From the neuroradiological point of view, only Pt1 and Pt2 displayed an MRI evocative of iron deposition. In contrast, Pt4 MRI only showed dysgenesis of the corpus callosum and prominence of the anterior commissure, but no signs of NBIA. Interestingly, Pt5 presented prenatal cortical and subcortical atrophy and pontocerebellar hypoplasia. Taking the clinical and neuroradiological features together, Pt5 shows a possible intermediate phenotype between CoPAN and PCH12, suggesting a continuum between the two disorders. Moreover, compared to cases reported in the literature,^6^, ^8^, ^9^, ^13^, ^14^, ^15^ our cases suggest that in contrast to PKAN, brain iron accumulation is not a prominent feature of *COASY*‐associated diseases. On the contrary, apart from cases with pure PHC12, different neuroradiological phenotypes can be observed, although a subtle genotype–phenotype correlation is not obvious.

Four of the *COASY* variants found in this work have never been described. The p.Arg377Leu, p.Glu408Lys, and p.Ile469Ter affect residues located in the DPCK functional domain, while the p.Pro260Ser falls in the PPAT domain. Apart from the p.Glu408Lys, which was found in the homozygous state (Pt1), all the other variants were in compound heterozygosity. Notably, no heterozygous compounds with both nonsense alleles were found, in line with previous reports associating the complete loss of COASY function with the PHC12 phenotype.^10^, ^11^ The p.Glu408Lys variant does not alter the amount of both mRNA and protein, and the site of the variant is moderately conserved. This site never hosts a positively charged residue like that introduced by the p.Glu408Lys, and the increase in vibrational entropy caused by the substitution is linked to a gain of molecular flexibility. However, the typical NBIA phenotype of Pt1 (PP4 criterion), the only patient showing the tiger's eye‐like sign, speaks for the likely pathogenicity of the variant, even if it remains classified as VUS.

Although COASY is the only known CoA synthase enzyme, no changes in CoA levels have been observed in fibroblasts from patients. However, we have recently reported normal levels of CoA in the brain of a conditional, neuronal‐specific *Coasy* null mouse model.^34^ Interestingly, several *PANK2* cellular models have not revealed any difference in total CoA levels.^35^ On the contrary, total CoA levels were found significantly decreased in fibroblasts derived from subjects harboring variants in both phosphopantothenoylcysteine synthetase (PPCS) and phosphopantothenoylcysteine decarboxylase (PPCDC), which catalyze the second and third steps of CoA *de novo* biosynthesis, respectively.^29^, ^36^ At present, we are not entirely clear why CoA levels are normal in fibroblasts derived from *COASY* patients. The first hypothesis could be related to the residual activity of the COASY protein. As we have already shown, fibroblasts from individuals with *COASY* variants that retain a residual amount of the protein have normal levels of CoA, although CoA *de novo* synthesis decreases significantly.^6^ These data indicate that the complete absence of CoA would likely be incompatible with life, as also suggested by the lethal phenotype caused by the complete loss of the COASY protein in the perinatal period in humans,^10^, ^11^ and in the embryonic period in mice.^34^ Furthermore, organisms have probably developed alternative strategies to counteract defects in *de novo* CoA biosynthetic pathway, which remain to be identified. A defect in PANK2 selectively disrupts the CoA‐dependent 4PPTylation of mitochondrial carrier proteins in the context of normal levels of CoA,^37^ leading to a reduction in the amount of these proteins in PKAN patient‐derived fibroblasts.^38^ Likewise, we found reduced amount of mtACP and ALDH1L2 proteins in the cells of all our *COASY* patients, while the transcripts levels were comparable to controls, suggesting a post‐translational regulation. These results reinforce the possible role of 4PPTylation as a common pathogenic mechanism in inborn errors of CoA metabolism, corroborating the pathogenicity of the new *COASY* variants here identified.

To identify possible common processes involved in the pathogenesis of the disease, we performed an unbiased bulk transcriptomic analysis. We found 982 deregulated genes in cells from patients compared to controls. Pathway enrichment analysis performed using different approaches revealed recurrent altered processes mainly related to mitochondrial bioenergetics, cell cycle and proliferation signaling, protein metabolism, and inflammation.

At first glance, it is not surprising that the mitochondria should be altered in a CoA‐related disorder since the mitochondria are the organelles with the highest CoA concentration.^39^ Accordingly, we found a consistent reduction of mitochondrial respiration in all our patients' cells. Interestingly, mtACP is not only an accessory subunit of mitochondrial complex I but is also involved in electron transfer, mitochondrial fatty acid synthesis required for lipoylation of target proteins, biogenesis of iron–sulfur clusters, and mitochondrial ribosome biogenesis and protein translation.^40^

Many biological processes identified in our transcriptome analysis are linked to the PI3K/Akt/mTOR pathway. This pathway triggers phospholipid production and is associated with protein synthesis, cell growth, and inflammation.^41^ Interestingly, a physical interaction between COASY and the ribosomal protein S6 kinase (S6K1), which is under the control of PI3K and mTOR, has been reported.^42^ In adipocytes, insulin stimulates S6K1‐dependent COASY protein phosphorylation, influencing lipid metabolism.^43^ COASY has been also demonstrated to form a functional complex with p85αPI3K, and it has been observed that COASY silencing alters the activity of the PI3K signaling pathway.^44^ Interestingly, a recent study showed that in response to hormones and growth factors, PI3K/AKT axis regulates CoA biosynthesis through direct phosphorylation of key enzymes, such as PANK2 and PANK4.^45^ Our data suggest a role of COASY in the regulation of cellular growth, as well as a possible PI3K/Akt/mTOR involvement in the pathogenesis of *COASY*‐associated diseases.

While the study acknowledges the limitations of using skin fibroblasts as a tissue to perform transcriptome analysis for a neurological and neurodevelopmental disorder, the findings lay the groundwork for future investigations using more relevant cell models. In this regard, studies are needed to verify and integrate these data in human iPSC‐derived neuronal cells and the brain of animal models.

To summarize, the description of five new patients carrying four novel variants of the COASY gene not only expands the genetic landscape of COASY‐related disorders but also reveals a variety of clinical features. The atypical manifestations, such as seizures and hearing loss, challenge the existing understanding of COASY‐associated diseases. The identification of epilepsy as a possible common feature opens new avenues for exploring the broader neurological impact of COASY variants. Our biochemical and molecular analysis suggests that, despite the heterogeneous clinical presentations, COASY‐associated disorders highlight common pathways that are also shared with other inborn errors of CoA metabolism.

## Conflict of Interest

The authors declare no conflict of interest.

## Author Contributions

I.D.M. and V.T.: conception and study design; all authors: data acquisition and analysis; I.D.M., V.T., and C.C.: text drafting and figures preparation.

## Data Availability

Datasets derived from this study are available at the repository Zenodo: https://zenodo.org/records/10261889 or from the corresponding author upon reasonable request.
